# The Impact of Normobaric Hypoxia and Intermittent Hypoxic Training on Cardiac Biomarkers in Endurance Athletes: A Pilot Study

**DOI:** 10.3390/ijms25094584

**Published:** 2024-04-23

**Authors:** Jakub Goliniewski, Miłosz Czuba, Kamila Płoszczyca, Małgorzata Chalimoniuk, Robert Gajda, Adam Niemaszyk, Katarzyna Kaczmarczyk, Józef Langfort

**Affiliations:** 1 Department of Sports Theory, The Jerzy Kukuczka Academy of Physical Education, 40-065 Katowice, Poland; golinmail@gmail.com (J.G.); j.langfort@awf.katowice.pl (J.L.); 2 Faculty of Rehabilitation, Józef Piłsudski University of Physical Education in Warsaw, 00-968 Warsaw, Poland; kamila.ploszczyca@gmail.com (K.P.); adam.niemaszyk@awf.edu.pl (A.N.); katarzyna.kaczmarczyk@awf.edu.pl (K.K.); 3 Department of Tourism and Health in Biała Podlaska, The Józef Piłsudski University of Physical Education, 00-968 Warsaw, Poland; mchalim@yahoo.com; 4 Department of Kinesiology and Health Prevention, Jan Dlugosz University, 42-200 Czestochowa, Poland; gajda@gajdamed.pl; 5 Center for Sports Cardiology at the Gajda-Med Medical Center in Pultusk, 06-100 Pultusk, Poland

**Keywords:** hypoxia, cardiac biomarkers, intermittent hypoxic training, endurance athletes

## Abstract

This study explores the effects of normobaric hypoxia and intermittent hypoxic training (IHT) on the physiological condition of the cardiac muscle in swimmers. Hypoxia has been reported to elicit both beneficial and adverse changes in the cardiovascular system, but its impact on the myocardium during acute exercise and altitude/hypoxic training remains less understood. We aimed to determine how a single bout of intense interval exercise and a four-week period of high-intensity endurance training under normobaric hypoxia affect cardiac marker activity in swimmers. Sixteen young male swimmers were divided into two groups: one undergoing training in hypoxia and the other in normoxia. Cardiac markers, including troponin I and T (cTnI and cTnT), heart-type fatty acid-binding protein (H-FABP), creatine kinase-MB isoenzyme (CK-MB), and myoglobin (Mb), were analyzed to assess the myocardium’s response. We found no significant differences in the physiological response of the cardiac muscle to intense physical exertion between hypoxia and normoxia. Four weeks of IHT did not alter the resting levels of cTnT, cTnI, and H-FABP, but it resulted in a noteworthy decrease in the resting concentration of CK-MB, suggesting enhanced cardiac muscle adaptation to exercise. In contrast, a reduction in resting Mb levels was observed in the control group training in normoxia. These findings suggest that IHT at moderate altitudes does not adversely affect cardiac muscle condition and may support cardiac muscle adaptation, affirming the safety and efficacy of IHT as a training method for athletes.

## 1. Introduction

Hypoxia is a state in which tissues, organs, or the whole body are deprived of adequate oxygen supply, which leads to a decline in exercise capacity [1,2]. Such alterations also impact directly on the myocardium. In both systemic and local hypoxia, the preference of the myocardium shifts to glucose as the primary substrate used for the resynthesis of adenosine triphosphate (ATP) [3]. Data obtained using animal models suggest that increased glucose metabolism is associated with adverse (pathological) changes in the myocardium [4,5]. It is assumed that this is a way of compensating for adverse changes in lipid metabolism, which often occur together with cardiomyocyte hypoxia [6]. The body’s response to insufficient oxygen supply also manifests itself in increased cardiac output, tachycardia, and significantly increased pulmonary blood pressure as a result of greater vascular resistance in the lungs [7].

On the one hand, therefore, these data suggest that exposure to hypoxia may induce certain adverse changes, perhaps of a transient nature, in the cardiovascular system, including the myocardium. On the other hand, however, there are also reports in the literature that indicate a beneficial effect of hypoxia on the physiological state of the heart. This is supported by the following observations: (1) lower mortality from heart diseases in people living in mountainous areas, with the drop in mortality progressively rising with the altitude at which study participants lived [8,9]; and (2) lower rates of heart disease risk factors and lower mortality from ischemic heart disease in men and women who reside at altitude compared with those living in lowland areas [10]. For instance, lower mortality from cardiovascular diseases, stroke, cancer, and Alzheimer’s disease has been reported in high-altitude regions in the Swiss [11] and Austrian Alps [9] and the western United States [8,11,12].

Nevertheless, there are no reports in the literature on the effects of a single bout of high-intensity exercise performed during acute exposure to hypoxia, or on the effects of altitude/hypoxic training in hypoxia, on the physiological state of the myocardium. Consequently, the response of the myocardium to hypoxia remains poorly studied, and available data are inconclusive.

In sports practice, altitude training making use of the hypoxia phenomenon has become a standard training protocol in many disciplines, with the aim of increasing exercise capacity at sea level, acclimatizing prior to competitions at altitude, or before ascending to altitude [13]. A sudden exposure of the human body to a hypoxic environment or a longer stay at elevated altitude induces numerous adaptations that can lead to athletes’ improved performance at sea level [14,15]. These mechanisms are generally attributed to either hematological [16], cardiovascular [7], or ventilatory [17] effects of altitude training. Such changes already occur in response to a 2–3 week stay at a moderate terrestrial altitude (2000–3000 m a.s.l.) or training in a normobaric hypoxia environment. Currently, the most popular strategies for enhancing the performance of athletes through exposure to hypoxia include intermittent hypoxic training (IHT) [18]. With IHT, athletes live at or near sea level, but train in hypoxia—either in natural high-altitude conditions or, more commonly, in artificial normobaric hypoxia. The results of previous studies indicate that exposure to hypoxia or physical exercise performed under these conditions can reduce the risk of cardiovascular diseases, as well as support the process of cardiac rehabilitation [19]. However, the rehabilitation process significantly differs from sports training in terms of intensity, training volume, and the training methods used. As such, little is so far known about the impact of high-intensity training conducted in hypoxic conditions on the physiological state of the cardiac muscle in athletes. 

The objective of this study, therefore, was to analyze the impact of a single bout of interval exercise and of sustained high-intensity endurance training, both in normobaric and hypoxic conditions, on the physiological state of the cardiac muscle in swimmers, as determined by the activity of cardiac markers: troponin I (cTnI) and T (cTnT), heart-type fatty acid-binding protein (H-FABP), creatine kinase-MB isoenzyme (CK-MB), and myoglobin (Mb). We posited the following hypotheses: (1) a single session of intensive interval exercise would contribute to an increase in cardiac marker activity, with hypoxic conditions not affecting the extent of these changes; and (2) a four-week high-intensity endurance training would lead to an adaptation of the cardiac muscle, resulting in decreased activity of cardiac markers, with a hypoxic environment indeed contributing to more profound changes in this area.

## 2. Results

### 2.1. The Effect of a Single Intense Interval Exercise Session in Normoxia and Hypoxia on the Concentration of Cardiac Markers in the Blood

**Figure 1:**
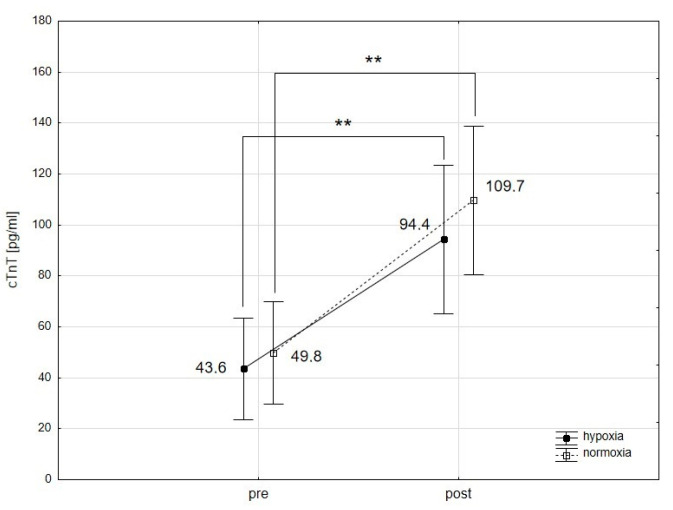
Troponin T (cTnT) levels before (pre) and after (post) a single intense interval exercise session in normoxia and hypoxia. ** *p* < 0.01 denote statistically significant differences (pre vs. post).

**Figure 2:**
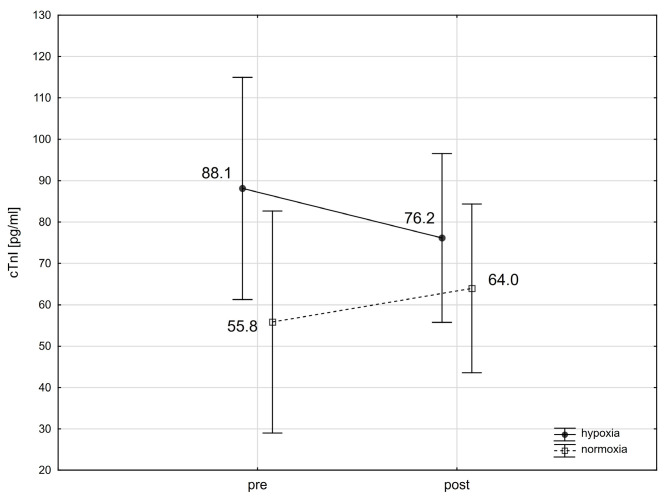
Troponin I (cTnI) levels before (pre) and after (post) a single intense interval exercise session in normoxia and hypoxia.

**Figure 3:**
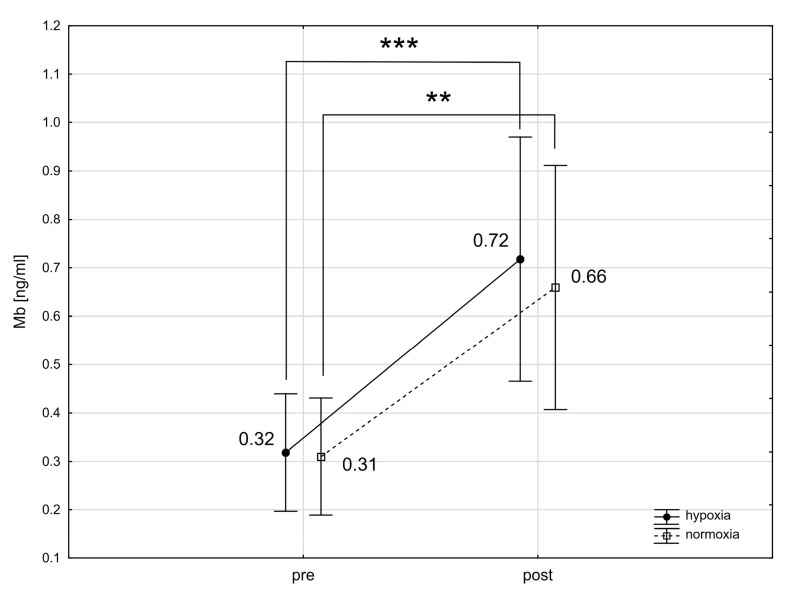
Myoglobin (Mb) levels before (pre) and after (post) a single intense interval exercise session in normoxia and hypoxia. *** *p* < 0.001, ** *p* < 0.01 denote statistically significant differences (pre vs. post).

**Figure 4:**
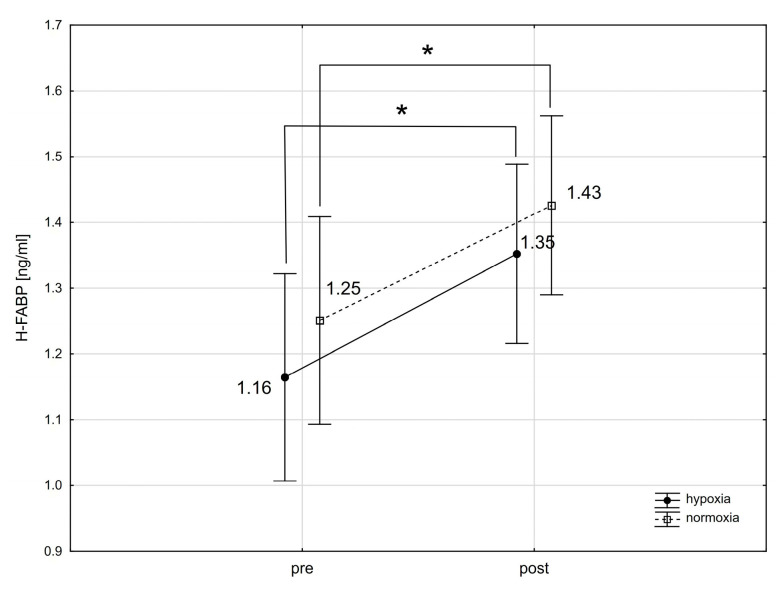
Heart-type fatty acid-binding protein (H-FABP) levels before (pre) and after (post) a single intense interval exercise session in normoxia and hypoxia. * *p* < 0.05 denote statistically significant differences (pre vs. post).

**Figure 5:**
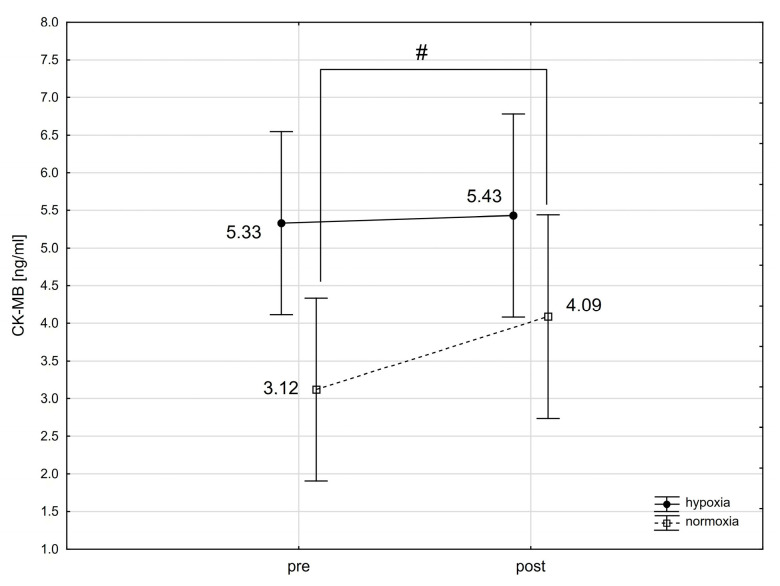
Creatine kinase-MB isoenzyme (CK-MB) levels before (pre) and after (post) a single intense interval exercise session in normoxia and hypoxia. # *p* < 0.07 pre vs. post.

The analysis using repeated-measures ANOVA showed a statistically significant impact of exercise on the levels of cTnT (F = 30.64; *p* < 0.001), Mb (F = 35.65; *p* < 0.001), CK-MB (F = 4.18; *p* < 0.05), and H-FABP (F = 17.29; *p* < 0.001). Subsequent Tukey’s post-hoc testing revealed a statistically significant rise in cTnT levels in the blood immediately following exercise, with an increase of 120.3% in normoxia (*p* < 0.01; ES: 1.16) and 116.5% in hypoxia (*p* < 0.01; ES: 1.11) (Figure 1). cTnI levels did not exhibit significant changes immediately after the exercise in either condition (Figure 2). Notably, Mb levels in the blood significantly rose by 112.9% (*p* < 0.01; ES: 1.55) after exercising in normoxia and by 125.0% (*p* < 0.001; ES: 0.80) in hypoxia (Figure 3). Similarly, H-FABP levels significantly increased immediately after exercise by 14.4% (*p* < 0.05; ES: 0.64) in normoxia and by 16.4% (ES: 0.62) in hypoxia (Figure 4). Additionally, there was a noticeable trend towards an increase in CK-MB activity by 31.1% (*p* < 0.07; ES: 0.46) after exercise in normoxia (Figure 5). The analysis did not reveal any statistically significant interactions between exercise and environmental conditions on the concentrations of the cardiac markers studied.

### 2.2. The Effect of Training in Normoxia and Hypoxia on the Resting Concentration of Cardiac Markers in the Blood

Our analysis identified a significant interaction between the group and training for the resting levels of CK-MB (F = 7.98; *p* < 0.05). IHT led to a notable decrease in resting CK-MB activity by 55.2% (*p* < 0.001; ES: 1.98). Such changes were not observed in the N group, which underwent training exclusively under normoxic conditions. The analysis also showed a significant training effect on the resting levels of Mb (F = 6.39; *p* < 0.05). Subsequent post-hoc analysis highlighted a significant reduction in resting Mb levels by 50.0% (*p* < 0.05; ES: 1.23) in the N group after undergoing training in normoxia. The resting levels of cTnT, cTnI, and H-FABP remained unchanged due to training, independent of the environmental conditions (Table 1).

### 2.3. The Effect of Training in Normoxia and Hypoxia on the Response of Cardiac Markers to Interval Exercise

**Figure 6:**
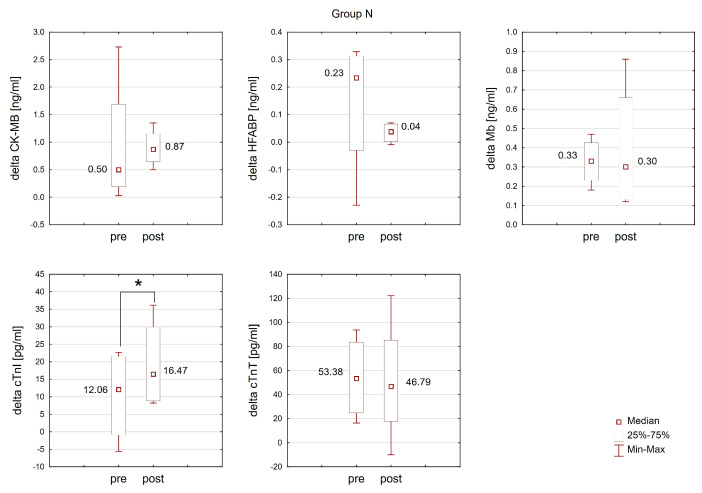
The response of cardiac markers to interval exercise before (pre) and after (post) training under normoxic conditions (Group N). * *p* < 0.05 denotes statistically significant differences when compared to the values measured before training.

**Figure 7:**
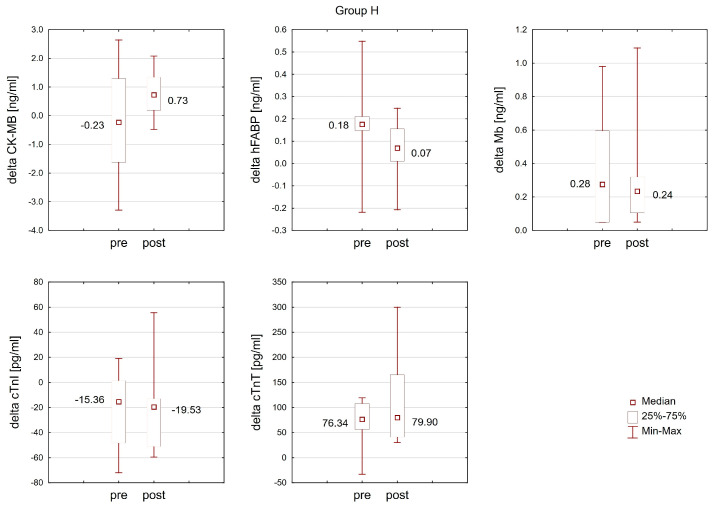
The response of cardiac markers to interval exercise before (pre) and after (post) training under hypoxic conditions (Group H).

The Wilcoxon matched-pairs test revealed that the change (delta) in the cTnT concentration after interval exercise in normoxia was significantly more pronounced (T = 3.00; *p* < 0.05; ES: 0.73) post-training in normoxia (Group N) compared to the change observed before training. No analogous changes were detected post-training in hypoxia (Group H). Moreover, training did not result in statistically significant changes in the response of other analyzed cardiac markers to interval exercise in either the N or H groups (Figure 6 and Figure 7).

## 3. Discussion

This study aimed to assess whether a single session of intense endurance exercise under normobaric hypoxia, as well as endurance training under normobaric hypoxia, impacts the physiological state of the heart in trained swimmers differently than training in normoxic conditions. We found no significant differences in the physiological response of the cardiac muscle to intense physical exertion between hypoxia and normoxia, suggesting that moderate hypoxia conditions (FiO_2_ = 15.5%) may not negatively affect the cardiac muscle in trained athletes. Additionally, our findings indicate that a 4-week regimen of IHT does not alter the resting levels of cTnT, cTnI, and H-FABP. However, a noteworthy decrease in the resting concentration of CK-MB post-IHT suggests enhanced cardiac muscle adaptation to exercise. Furthermore, while no changes were noted in the resting Mb concentration in the blood post-training in the hypoxia group, a reduction was observed in the control group.

Early studies of the effects of exercise on cardiac marker concentrations in athletes focused mainly on prolonged exercise [20,21,22], and most publications confirmed elevated levels of the markers being tested after competitions or endurance tests. In recent years, interest in this topic has extended to include the impact of short-duration high-intensity exercise. However, as yet, there are not many studies in this area, and their reported findings are contradictory: some publications confirm elevations in cardiac enzymes following this type of exercise [23,24,25], whereas others have not confirmed this effect [26,27]. This discrepancy can be explained through cardiac diagnostics, namely, measurements of serum concentrations of cardiac biomarkers [28,29].

The response of cardiac markers post-exercise varies depending on the type, duration, and intensity of the activity [30]. Notably, the high-intensity endurance training employed in our study more effectively activates cardio-specific markers [31] and leads to more significant adaptive changes than moderate-intensity training [32]. It is believed that the release of cardiac markers is directly affected not only by the training workload but also by the environmental conditions in which the exercise is performed, including altitude above sea level [33]. It remains unclear how training under hypoxic conditions affects myocardial function. Our study contributes new insights into this area.

### 3.1. Troponins (cTnT and cTnI)

We observed a significant increase in cTnT levels following intense physical exertion under both hypoxic and normoxic conditions. cTnI levels, however, did not significantly change under either condition. Li et al. [33] noted an increase in cTnT immediately after exercise in both normoxia and hypoxia, with a significant rise in cTnI only 2 and 4 h after exercise in hypoxic conditions. Klinkenberg et al. [34] found that cTnT reached its peak after 2 h, while cTnI peaked 5 h after the completion of endurance exercise. Similarly, Skadberg et al. [35] reported that cTnT and cTnI peaked immediately and 3 h post-exercise, respectively. These findings indicate that cTnI and cTnT respond differently to physical exercise, with cTnI demonstrating a slower release. Note that in our study, troponin activity was measured only immediately after exercise, which might explain the absence of significant changes in cTnI activity. Furthermore, studies have linked the length of training to the release of cardiac troponins during strenuous physical activity [36,37]. Athletes with more extended training histories exhibit a slower release of troponins compared to those with shorter histories, which could also influence the results of our study. The athletes in our study were highly trained, which may have slowed the release of cTnI and cTnT post-exercise due to long-term adaptation to physical training.

Our results show that four weeks of training had no impact on resting troponin levels, regardless of whether the exercise had been conducted under normoxic or hypoxic conditions. These findings partly corroborate earlier reports suggesting that the increase in troponin activity in response to training mainly depends on the exercise’s volume and intensity, and only secondarily on the degree of hypoxia. Mellor et al. [38] measured cardiac markers during a 10-day high-altitude trek (from 3833 m to 5129 m above sea level), where participants engaged in long hikes at different altitudes, interspersed with rest days. Increases in cTnT levels were noted both at the lowest and highest altitudes, with no changes observed at intermediate altitudes. The physical strain was highest on the first and last days of the expedition, with intermediate altitude exertions deemed low by participants and not affecting cTnT levels. Our findings suggest that IHT at moderate altitudes does not alter resting troponin levels, offering a safe alternative to normoxic condition training in this respect.

In our study, the post-exercise increase in cTnI activity was found to be more significant after normoxic condition training (Group N) than before training began. The experimental group training under hypoxia did not show similar changes. Such differences could be attributed to the lesser absolute workload, which imposed less strain on participants in the experimental group. Interestingly, after a single session of interval exercise in hypoxia, both before and after the training program, there was a visible downward trend in cTnI levels, while in normoxia, cTnI activity increased. This suggests the possibility of a mechanism that reduces activity or slows the kinetics of cTnI concentration changes under hypoxia, warranting further research. Many authors [39,40,41] also emphasize significant individual variation in the post-exercise release of troponins, as was evident in our study as well.

### 3.2. Cardiac Isoenzyme of Creatine Kinase (CK-MB)

Previous research on the CK-MB response to physical exercise has shown an increase in CK-MB levels during and after both prolonged endurance activities [37,42], short bouts of high-intensity effort [24,27], and resistance training [43]. However, there have been a few instances where studies reported no change or even a reduction in CK-MB activity post-exercise compared to resting levels [44]. Our study found that CK-MB levels remained largely unchanged after a single session of exercise in hypoxia, while exercise in normoxia resulted in a slight increase in CK-MB (*p* < 0.07). These variations could be attributed to the different relative loads applied in interval exercises. In our study groups, the relative load (%VO_2max_) was tailored to the specific conditions (normoxia vs. hypoxia; refer to the methodology section). Consequently, in hypoxic conditions, participants engaged in mechanically less demanding work (approximately 10% lighter), which could explain the diminished CK-MB response to the exercise [45,46]. Moreover, the significant difference in baseline CK-MB concentrations before exercising in hypoxic and normoxic conditions (5.33 vs. 3.12 ng/mL) could have influenced the observed post-exercise changes in CK-MB levels. 

Training under normoxic conditions did not alter the resting CK-MB levels. Interestingly, in the group training under hypoxia, we observed a reduction in resting CK-MB activity compared to their baseline values. This could be due to an enhanced adaptation to the training stimuli under hypoxic conditions. However, our training regimen did not significantly impact the degree of change in CK-MB activity post-exercise in either group, irrespective of the training environment. Here, it is worth noting the results of animal studies analyzing the effects of 4 weeks of continuous and intermittent hypoxia exposure (23 and 8 h per day) on the levels of various creatine kinase isoforms, including mitochondrial (mtCK) [47]. The observed increase in total CK activity, particularly in the left ventricle, was primarily linked to mtCK expression, suggesting adaptive changes due to hypoxia—mtCK is involved in oxidative phosphorylation, enhancing ADP availability, affecting membrane potential regulation, and reactive oxygen species production [48]. Subsequent research in this direction seems to support the crucial role of the mitochondrial isoform, with its overexpression providing protective benefits to heart cells [49]. These reports seem to point to potential limitations in our study concerning CK-MB activity, due to the possible distortion of results by the immunoinhibition method, which may mistakenly recognize mtCK as CK-MB [50]. Therefore, future studies should focus significantly on the mitochondrial isoform of creatine kinase.

### 3.3. Heart-Type Fatty Acid-Binding Protein (H-FABP)

Heart-type fatty acid-binding protein (H-FABP) serves as another diagnostic marker for cardiac muscle, yet research on how physical exercise affects its levels is scarce. H-FABP plays a crucial role in the heart by transporting fatty acids to the mitochondria for β-oxidation, leading to energy release [51]. During cardiac ischemia, there is an increase in fatty acid levels in both plasma and cardiac muscle tissue, detrimental to the heart [52]. This, along with documented H-FABP leakage from cardiac tissue during ischemia [53], underscores the protein’s vital role in safeguarding cardiac cells. H-FABP is also believed to act as a scavenger of free radicals generated during ischemia [54]. Despite being regarded as cardio-specific due to its abundance in cardiac muscle [55], it should be borne in mind that H-FABP is also found in lower concentrations in skeletal muscles [56].

The number of studies on the impact of physical exercise on H-FABP is limited. Sorichter et al. [57] reported an increase in FABP (without specifying its cardiac fraction) after a 20 min downhill run at 70% VO_2max_ intensity. A similar increase was observed during a 21 km time trial run at submaximal intensity [58]. However, such H-FABP changes were not observed in exercise tests involving individuals with coronary artery disease [59,60]. Our study revealed an increase in H-FABP concentration after a single intense exercise session in both hypoxia and normoxia among athletes, with no difference in the magnitude of these changes between conditions. The maximum H-FABP concentration did not exceed the normal range for healthy individuals [61], which, combined with the previously mentioned studies, suggests that the increase could be attributed to H-FABP leakage from skeletal muscles.

We did not observe significant changes in either resting or post-exercise H-FABP activity following training. Note that the effects of physical training on H-FABP levels are not fully understood, with existing research yielding conflicting results [62,63]. These discrepancies stem largely from variations in research methodology, such as the duration of the training period and the intensity of the exercises. Sponder et al. [63] found a reduction in resting H-FABP levels after 8 months of training in healthy adults aged 30–65 years, attributed to enhanced cardiac muscle perfusion from training adaptation, thus diminishing H-FABP leakage into the extracellular space, suggesting a cardioprotective benefit of physical exercise. On the other hand, Saheed et al. [62] reported no changes in resting H-FABP levels after a 12-week training period in a similar group, aligning with our results. Our study included athletes with a high level of training adaptation, engaged in years of sports training, which likely limited post-training adaptations in cardiac muscle physiology and led to no changes in H-FABP activity from the training program.

### 3.4. Mioglobin (Mb)

Early research on the impact of physical exercise on post-exercise Mb concentration highlighted the duration of the exercise [64], the training history, and the age of participants [65] as significant factors differentiating the Mb response. Increases in Mb have been noted following high-intensity interval exercises, endurance activities [66], and resistance training [67]. Our findings align with these earlier observations. The post-exercise surge in Mb concentration is directly linked to sarcolemma damage from muscle activity, leading to sodium–potassium pump dysfunction and the release of various metabolites and intracellular proteins, including Mb, into the bloodstream [68]. In severe cases, this can result in exercise-induced rhabdomyolysis [69].

Initial findings suggested that hypoxic conditions might contribute to an increase in Mb concentration [70]. Nonetheless, more recent investigations have shown that a supplementary factor, such as physical exercise, is required for such an increase, and the duration of hypoxia exposure must extend beyond a single session [71]. Shave et al. [72] found that Mb levels did not significantly rise in triathletes immediately after 120 min of cycling at an intensity near the anaerobic threshold, under both normoxic and hypoxic conditions (FiO_2_ = 15%). In our study, employing a very similar hypoxic stimulus (FiO_2_ = 15.5%, ~2500 m above sea level) and high-intensity interval exercise (90%VO_2max_), we noticed an increase in Mb levels in both normoxia and hypoxia right after exercise, with no significant difference in the extent of these changes between the two conditions. Our results, alongside previous studies [72], suggest that the primary factor affecting the rise in Mb levels post-exercise is its intensity, rather than the application of moderate hypoxic stress.

Following a four-week training period, a notable decrease in Mb levels (by 50%) was observed in the group training under normoxia (Group N). This contrasted with the hypoxia training group (Group H), where no similar changes were detected. These results are supported by prior research. Jacobs et al. [73] observed a lower Mb concentration following a six-week high-intensity training cycle conducted in normoxia. In a study by Terrados et al. [74], comparisons were made between lower limbs, with one trained under normoxic conditions and the other under hypoxia (2300 m above sea level), showing that a four-week training cycle resulted in a decrease in myoglobin levels in the normoxia-trained leg and an increase in the hypoxia-trained limb. In research examining the effects of an eight-week training cycle under hypoxia (2500 m above sea level) and normoxia on Mb levels, no changes were noted in skeletal muscles despite a considerable improvement in the aerobic capacity of the participants [75].

Our study found that, in Group H, the resting Mb concentration was unaffected by the training program. This outcome might indicate stimulation of Mb expression by hypoxia. It appears that the combination of hypoxic conditions, which promote calcium release from the endoplasmic reticulum, and physical exercise, supporting the calcium/calcineurin/NFAT pathway, significantly boosts NFAT translocation to the nucleus and enhances Mb expression [71,76].

A significant challenge and limitation in interpreting our results lies in differentiating the source of Mb, essential for evaluating the impact of hypoxia on the circulatory system. Currently, the scientific literature is mainly restricted to animal studies. Nicholson et al. [77] conducted research on rats undergoing a four-week training cycle with voluntary physical activity. An anticipated increase in Mb was observed solely in skeletal muscles, with a reduction in cardiac muscle. This outcome was attributed to decreased NFAT activity due to physical training, where the CN/NFAT signaling pathway governs Mb expression. Yet, detailed studies on the specific mechanisms by which physical exercise influences its regulation are still lacking. The authors also noted their findings might not translate to larger mammals. Heart expression studies were carried out using rat heart homogenates, complicating the application of this method to human hearts.

### 3.5. Study Limitations

In our study, for technical reasons, blood samples to measure cardiac markers were taken only before the start of the exercise and immediately upon its completion. We did not track the kinetics of changes in the examined biomarkers throughout the post-exercise period at multiple measurement points. Future research in this field should aim to broaden the scope to include a longer training duration and, most importantly, to explore the kinetics of cardiac marker activity over an extended timeframe. This approach could potentially enable the verification of the conclusions made in this study. To gain a deeper understanding of the potential mechanisms at play in the heart under hypoxic conditions, it will also be advisable to incorporate additional blood markers, such as natriuretic peptide or copeptin, into diagnostic efforts. Additionally, this study was limited to male participants with similar levels of training experience. Future research should, therefore, expand to include female participants and individuals with varied levels of training experience.

## 4. Materials and Methods

### 4.1. Participants

This study involved 16 young male well-trained swimmers (aged 18 to 25 years) participating in swimming competitions. The inclusion criteria required a minimum of a six-month washout period from any previous altitude training and baseline blood cardiac markers within the reference range. Exclusion criteria included serious injury, medications, dehydration, and infection found during the entire experiment. None of the participants were excluded from the project. The characteristics of the study group are presented in Table 2. 

Participants were randomly assigned to two equal-sized groups. Group H (*n* = 8) followed their training regimen, plus three high-intensity IHT sessions per week. Group N (*n* = 8) performed an identical program, albeit in normoxia.

All participants had current valid medical examinations confirming they were in good health and were medically permitted to engage in sporting activity. Before commencing the study, all participants were informed about the purpose and course of the study and provided their written consent to participate. The research project was conducted according to the Helsinki Declaration and was approved by the Bioethics Committee at the University of Zielona Góra, Poland (Resolution No. 21/2022 of 9 November 2022).

### 4.2. Study Design

This study sought to examine the impact of a sudden bout of normobaric hypoxia and intermittent hypoxia training on the activity of cardiac markers. To this end, apart from their regular swim training regimen in normoxia, group H also performed high-intensity IHT three times a week. Group N, on the other hand, performed an identical training program (swimming regimen + training in the lab) entirely in normoxia. Two series of tests were conducted at the Hypoxia Lab at the Jerzy Kukuczka Academy of Physical Education in Katowice, Poland. The first series (baseline measurements) was performed before the training program and during the first dryland training sessions (in either normoxia or hypoxia). The second series of tests were carried out during the final dryland training session, concluding the 4-week training program.

### 4.3. Measures

The first test series (S1) began with measurements of height, mass, and body composition (InBody 220, Biospace, Seoul, Republic of Korea) in the morning, after an overnight fast. Then, 2 h after consuming a light meal, a ramp test on an Excalibur Sport cycle ergometer (Lode, Groningen, The Netherlands) was performed to determine the maximum oxygen uptake (VO_2max_) and establish training loads (%VO_2max_). The exercise test began with a load of 30 W, which further increased by 25 W every minute. The test was performed until voluntary exhaustion (the participant was unable to sustain the load). During the test, heart rate (HR), breathing frequency (BF), oxygen uptake (VO_2_), minute ventilation (VE), and the amount of exhaled carbon dioxide (VCO_2_) were monitored using a MetaMax 3B ergospirometry set (Cortex, Leipzig, Germany). On the second day of testing, after 24 h of passive rest, all participants repeated the ramp test under normobaric hypoxia conditions (altitude 2500 m a.s.l.) to determine relative loads (%VO_2max_hyp) for IHT. During S1, before and immediately after the first two training sessions (conducted under normoxia and hypoxia conditions), venous blood was drawn from the cubital vein to determine the impact of interval exercise on the activity of cardiac markers cTnT and cTnI, H-FABP, Mb, and CK-MB. After completing S1, participants continued the 4-week training program in hypoxia (group H) and normoxia (group N). The second test series (S2) was performed during the last training session under conditions in which the respective groups trained, and venous blood was drawn to again assess changes in cardiac marker levels, according to the same protocol as for S1.

### 4.4. Training Protocol

A 4-week training program with an increasing training load was designed for the purposes of this study. Each week, participants of groups H and N performed 2 interval training sessions in a laboratory equipped with a normobaric hypoxia generation system (LOS-HYP1/3NU, Lowoxygen Systems, Berlin, Germany). Training sessions for group H were conducted under simulated altitude conditions of 2500 m a.s.l. (FiO_2_ = 15.5%), while participants in group N performed the same training in the same laboratory but under normoxic conditions. Participants were not informed under which conditions the laboratory sessions were conducted. For each participant, the load was adjusted based on %VO_2max_ (group N) or %VO_2max_hyp (group H). Each training session consisted of a 10 min general warm-up, a 45–55 min main part, and a 10 min cool-down. The main part consisted of exercise performed on an upper limb rotator (50 W) with a cadence of 80 rpm lasting 60 s. Then, the swimmers performed a 30 s maximum effort on the rotator from a flying start with a load of 0.4 Nm·kg^−1^ (Brachumera Sport, Lode, Groningen, The Netherlands). After a 30 s rest period, the athletes performed a 3 min ride on a cycle ergometer (Cyclus 2) with the intensity set at 50% VO_2max_hyp/VO_2max_. Following a three-minute period of active rest on the cycle ergometer, the swimmers performed another two-minute bout of exercise, at an intensity of 95% VO_2max_hyp/VO_2max_. After the high-intensity phase of training for the lower limbs, the athletes peddled for three minutes at the intensity of 50% VO_2max_hyp/VO_2max_. This circuit was repeated four times in the first four interval training sessions, after which a fifth circuit was added to increase the overall training load. The swimming training was the same for athletes in both the normoxic and hypoxic groups with a volume of approximately 50 km per week (Table 3).

For the biochemical assays, we utilized an ELISA ELx 800 BioTek reader (Swindon, UK). Blood samples underwent centrifugation to separate serum, which was subsequently stored at −80 °C for subsequent marker analysis. The markers analyzed included cardiac troponin T (Human Troponin T cardiac muscle, Wuhan EIAab Science Co., Ltd., Wuhan, China), cardiac troponin I (Human Troponin I cardiac muscle ELISA kit, Wuhan EIAab Science Co., Ltd., Wuhan, China), myoglobin (Human Myoglobin, Wuhan EIAab Science Co., Ltd., Wuhan, China), the MB isoenzyme of creatine kinase (Test Kit BC-1121, Biocheck Inc., Foster City, CA, USA), and heart-type fatty acid-binding protein (Human FABP ELISA KIT SIGMA-ALDRICH). Troponin levels were measured using an immunoassay, while H-FABP levels were determined by a colorimetric method. Adjustments for post-exercise plasma volume changes (ΔPV%) in the analysis of all biomarkers were based on van Beaumont’s formula (1972).

### 4.5. Statistical Analysis

The statistical analysis was performed using Statistica v.13 (StatSoft). To characterize the structure of the variables studied, basic descriptive statistics were calculated as follows: arithmetic mean (*x*), standard deviation (SD), median (Me), and lower and upper quartiles (Q1 and Q3). Normality of distribution was assessed using the Shapiro–Wilk test. Homogeneity of variances was tested with Levene’s test. To verify the significance of differences between successive measurements under normoxia and hypoxia (effort × conditions), and between groups (group × training), a two-way ANOVA with repeated measures was used. If significant differences were found, further analysis was conducted with Tukey’s post-hoc test. Due to the lack of normality in the distribution of variables, the significance of differences for the magnitude of changes (delta) in cardiac marker activity in the blood under the influence of interval exercise in normoxia and hypoxia before and after four weeks of training was checked with the Wilcoxon matched-pairs test, separately for the N and H groups. For all analyses, a statistical significance level of *p* < 0.05 was adopted. Additionally, to determine the effect size (ES), Cohen’s *d* indicator was used, and the following ranges for ES were used in interpreting the results: small (0.20–0.60), moderate (0.60–1.20), large (1.20–2.0), very large (2.0–4.0), or extremely large (>4.0) [78].

## 5. Conclusions

Our investigation examined the impact of moderate normobaric hypoxia (2500 m), combined with either a single bout of intense exercise or IHT, on cardiac markers in endurance athletes.

Firstly, our findings did not demonstrate a significant increase in cardiac marker activity following a single bout of intense interval exercise under hypoxia, paralleling the responses observed under normoxic conditions. This observation suggests that acute exposure to moderate hypoxia does not exacerbate the myocardial stress response to high-intensity exercise in well-trained athletes, thus not confirming our first research hypothesis, positing a potential elevation in cardiac marker activity due to the hypoxic stimulus.

Moreover, the adaptations we expected to see in cardiac marker activity following a four-week period of IHT were only partially observed. While the IHT did not significantly alter the resting levels of cTnT, cTnI, and H-FABP, consistent with our second hypothesis’s expectation of decreased cardiac marker activity indicative of myocardial adaptation, a noteworthy reduction in resting CK-MB levels was observed. This finding suggests a complex interplay of factors governing myocardial adaptation to hypoxia, which may not be fully captured through the activity of these markers alone.

In sum, our findings indicate that neither a single session of exercise performed under hypoxia, nor a four-week regimen of IHT training under hypoxia, lead to an increase in cardiac marker activity in swimmers, in comparison to the changes observed under normoxic conditions. 

While our study confirms the safety and potential benefits of IHT for cardiac muscle adaptation in endurance athletes, it also highlights the need for further research to fully understand the mechanisms underpinning these adaptations. Specifically, future studies should aim to elucidate the role of different hypoxic training modalities and their intensity on cardiac health and performance in athletes. Additionally, expanding the range of cardiac markers analyzed and including longer-term follow-up assessments may provide a more comprehensive understanding of the myocardial adaptations to hypoxic training and its implications for athlete health and performance optimization.

## Figures and Tables

**Table 1:** Resting values of cardiac markers before and after training under hypoxic (Group H) and normoxic (Group N) conditions. *** *p* < 0.001, * *p* < 0.05 denote statistically significant differences when compared to the values measured before training.

	Group H		Group N	
	Before	After	Before	After
cTnT (pg/mL)	44.4 ± 33.6	60.8 ± 31.5	44.8 ± 39.7	29.3 ± 18.5
cTnI (pg/mL)	125.9 ± 85.9	159.4 ± 69.6	43.7 ± 12.6	114.3 ± 67.7
Mb (ng/mL)	0.26 ± 0.14	0.23 ± 0.18	0.36 ± 0.18	0.18 ± 0.09 *
H-FABP (ng/mL)	1.17 ± 0.35	1.06 ± 0.23	1.26 ± 0.24	0.18 ± 0.09 *
CK-MB (ng/mL)	4.75 ± 1.70	2.13 ± 0.77 ***	3.15 ± 2.21	2.52 ± 1.68

**Table 2:** Characteristics of the subject.

Variable	Group H (*n* = 8)	Group N (*n* = 8)
Age (years)	19.1 ± 1.3	20.5 ± 1.3
Body height (m)	1.83 ± 0.03	1.81 ± 0.04
Body mass (kg)	76.4 ± 5.4	74.1 ± 6.3
Body fat (%)	9.3 ± 3.5	9.5 ± 1.6
VO_2max_ (mL/kg/min)	56.0 ± 4.0	53.7 ± 5.8
WR_max_ (W)	362.0 ± 22.2	352.7 ± 33.1
P_peak_ (W)	741.0 ± 124.3	784.6 ± 149.5
P_mean_ (W)	452.7 ± 37.6	435.1 ± 35.4

VO_2max_—maximal oxygen uptake during the ramp test, WR_max_—maximal workload during ramp test, P_peak_—peak power during Wingate test for upper limbs, P_mean_—mean power during Wingate test for upper limbs.

**Table 3:** Training program during the experiment.

Day	Microcyle 1	Microcyle 2	Microcyle 3	Microcyle 4
1	(1) AM: 1 h—TL4 + 30 min swimming REC (2) PM: 2 h—swimming EN2	(1) AM: 1 h—TL4 + 30 min swimming REC (2) PM: 2 h—swimming EN2	(1) AM: 1:15 h—TL5 + 45 min swimming REC (2) PM: 2 h—swimming EN2	(1) AM: 1:15 h—TL5 + 45 min swimming REC (2) PM: 2 h—swimming EN2
2	(1) AM: 1 h—ST + 1 h EN3 swimming (2) PM: 2 h—swimming EN2	(1) AM: 1 h—ST + 1 h EN3 swimming (2) PM: 2 h—swimming EN2	(1) AM: 1 h—ST + 1 h EN3 swimming (2) PM: 2 h—swimming EN2	(1) AM: 1 h—ST + 1 h EN3 swimming (2) PM: 2 h—swimming EN2
3	(1) AM: 2 h—SP1 swimming (2) PM: off	(1) AM: 2 h—SP1 swimming (2) PM: off	(1) AM: 2 h—SP1 swimming (2) PM: off	(1) AM: 2 h—SP1 swimming (2) PM: off
4	(1) AM: 1 h—TL4 + 30 min swimming REC (2) PM: 2 h—swimming EN2	(1) AM: 1 h—TL4 + 30 min swimming REC (2) PM: 2 h—swimming EN2	(1) AM: 1:15 h—TL5 + 45 min swimming REC (2) PM: 2 h—swimming EN2	(1) AM: 1:15 h—TL5 + 45 min swimming REC (2) PM: 2 h—swimming EN2
5	(1) AM: 1 h—ST + 1 h EN3 swimming (2) PM: 2 h—swimming EN2	(1) AM: 1 h—ST + 1 h EN3 swimming (2) PM: 2 h—swimming EN2	(1) AM: 1 h—ST + 1 h EN3 swimming (2) PM: 2 h—swimming EN2	(1) AM: 1 h—ST + 1 h EN3 swimming (2) PM: 2 h—swimming EN2
6	(1) AM: 2 h—SP1 swimming (2) PM: off	(1) AM: 2 h—SP1 swimming (2) PM: off	(1) AM: 2 h—SP1 swimming (2) PM: off	(1) AM: 2 h—SP1 swimming (2) PM: off
7	off	off	off	off

TL4—Training in the lab—4 circuits, TL5—training in the lab—5 circuits, ST—core stability training, REC—recovery training (up to 75% HR_LT_), EN2—endurance training (75–85% HR_LT_), EN3—endurance training (95–105% HR_LT_), SP1—anaerobic capacity training.

## Data Availability

The data presented in this study are available on request from the corresponding author.

## Abbreviations

The list of abbreviations used in the publication:

**Statistical analysis**	
ES	the effect size
F	F-value
Me	median
p	*p*-value
Q1	lower quartiles
Q3	upper quartiles
SD	standard deviation
x	arithmetic mean
**Cardiac biomarkers**	
CK-MB	creatine kinase-MB isoenzyme
CN/NFAT	calcineurin nuclear factor of activated T-cells
cTnI	troponin I
cTnT	troponin T
H-FABP	heart-type fatty acid-binding protein
MB	myoglobin
mtCK	creatine kinase isoforms, including mitochondrial
NFAT	nuclear factor of activated T-cells
**Physiological variables**	
BF	breathing frequency
FiO_2_	fraction of inspired oxygen
HR	heart rate
VCO_2_	exhaled carbon dioxide
VE	minute ventilation
VO_2_	oxygen uptake
VO_2max_	maximal oxygen uptake
VO_2max_hyp	maximal oxygen uptake in hypoxia
WR_max_	maximal workload
P_peak_	peak power during Wingate test for upper limbs
P_mean_	mean power during Wingate test for upper limbs
ΔPV%	plasma volume changes
**Training**	
EN2	endurance training (75–85% HR _LT_)
EN3	endurance training (95–105% HR _LT_)
IHT	intermittent hypoxic training
Pmean	mean power during Wingate test for upper limbs
Ppeak	peak power during Wingate test for upper limbs
REC	recovery training
S1	first test series
S2	second test series
SP1	anaerobic capacity training
ST	core stability training
TL4	training in the lab—4 circuits
TL5	training in the lab—5 circuits
